# Supplementary figures and images for: Systematic identification of 20S proteasome substrates
Source: Mol Syst Biol. 2024 Jan 29;20(4):403–27. doi: 10.1038/s44320-024-00015-y (PMC10987551; doi:10.1038/s44320-024-00015-y)

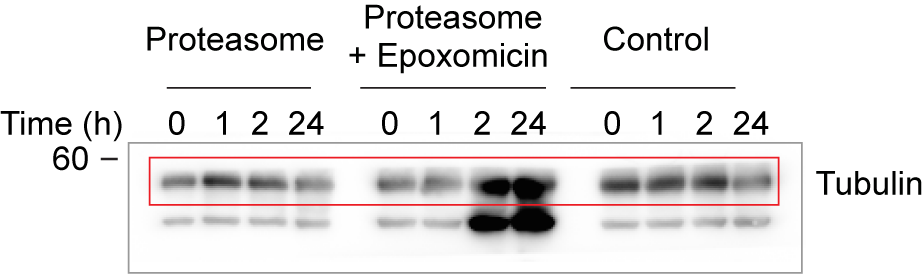

Supplement: Supplementary file 5 — Source Data Fig. 2 [file 44320_2024_15_MOESM5_ESM.zip › SD_Figure_2/2D/Fig 2D Western Tubulin.tif]

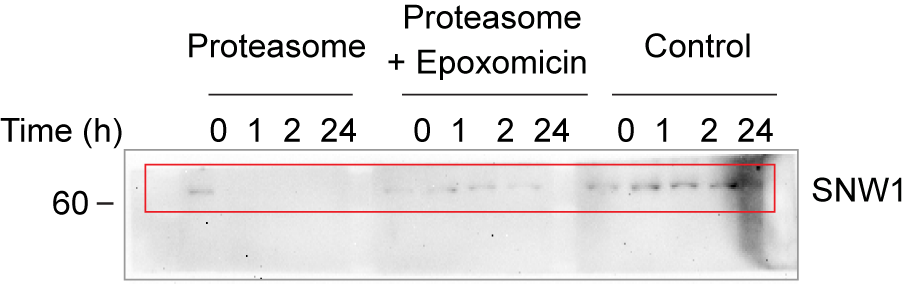

Supplement: Supplementary file 5 — Source Data Fig. 2 [file 44320_2024_15_MOESM5_ESM.zip › SD_Figure_2/2D/Fig 2D Western SNW1.tif]

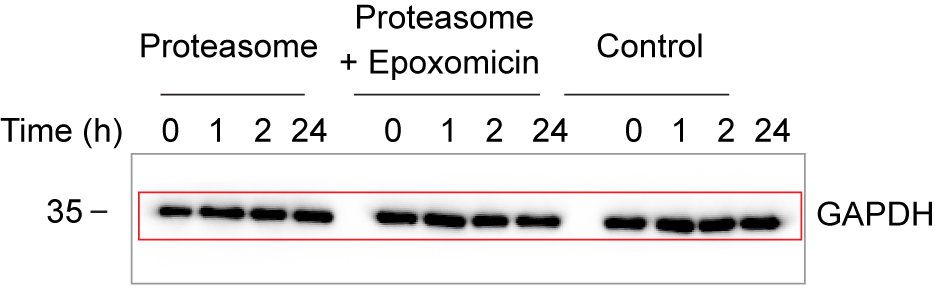

Supplement: Supplementary file 5 — Source Data Fig. 2 [file 44320_2024_15_MOESM5_ESM.zip › SD_Figure_2/2D/Fig 2D Western GAPDH.tif]

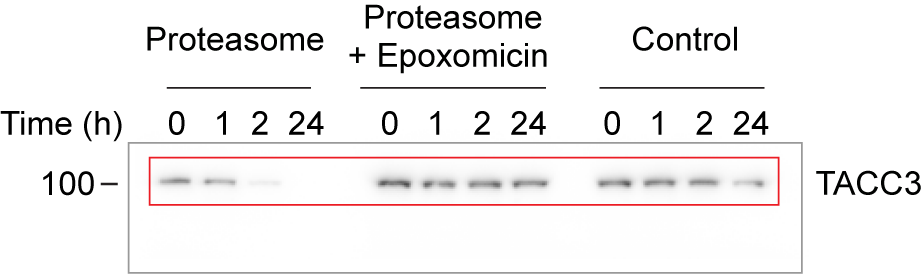

Supplement: Supplementary file 5 — Source Data Fig. 2 [file 44320_2024_15_MOESM5_ESM.zip › SD_Figure_2/2D/Fig 2D Western TACC3.tif]

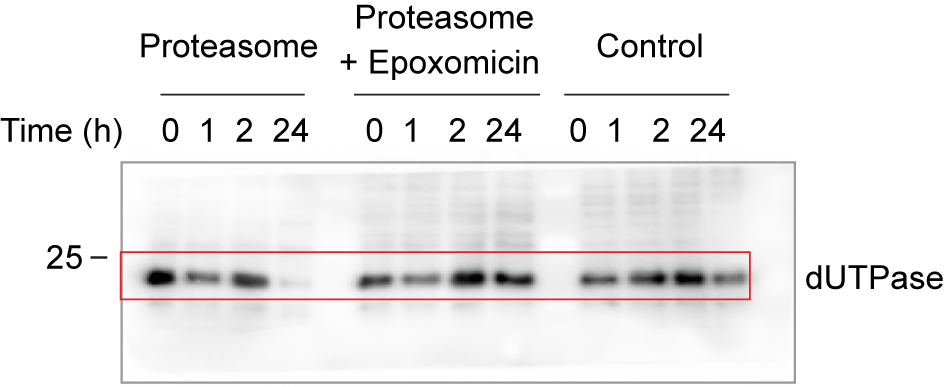

Supplement: Supplementary file 5 — Source Data Fig. 2 [file 44320_2024_15_MOESM5_ESM.zip › SD_Figure_2/2D/Fig 2D Western dUTPase.tif]

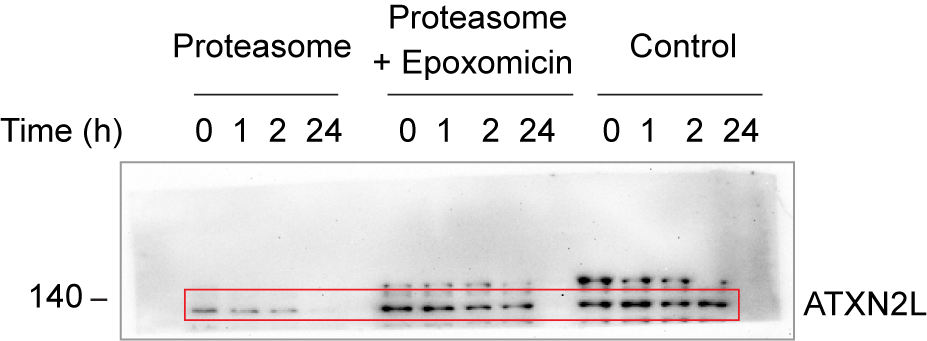

Supplement: Supplementary file 5 — Source Data Fig. 2 [file 44320_2024_15_MOESM5_ESM.zip › SD_Figure_2/2D/Fig 2D Western ATXNL2.tif]

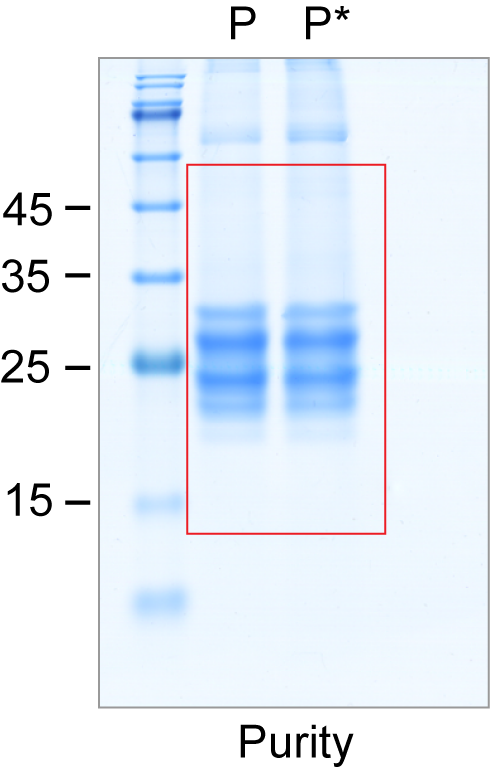

Supplement: Supplementary file 6 — Source Data Fig. 4 [file 44320_2024_15_MOESM6_ESM.zip › SD_Figure_4/4B/Fig 4B Coomassie 20S - Purity.tif]

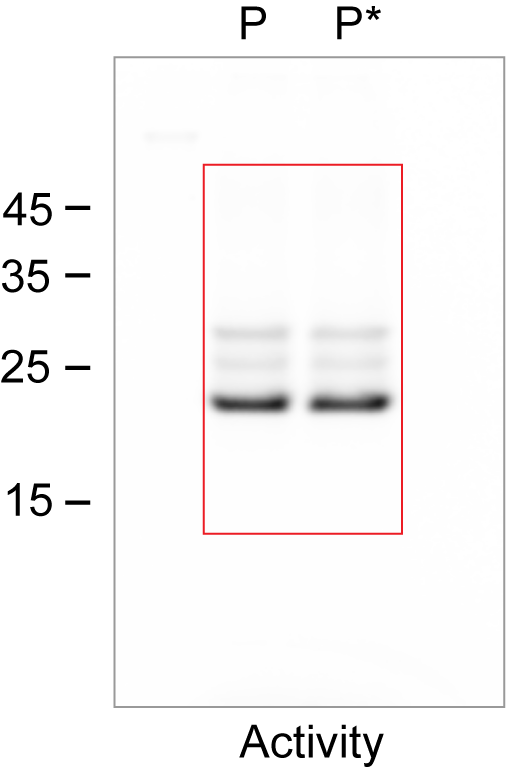

Supplement: Supplementary file 6 — Source Data Fig. 4 [file 44320_2024_15_MOESM6_ESM.zip › SD_Figure_4/4B/Fig 4B Fluorescence 20S - MV151.tif]

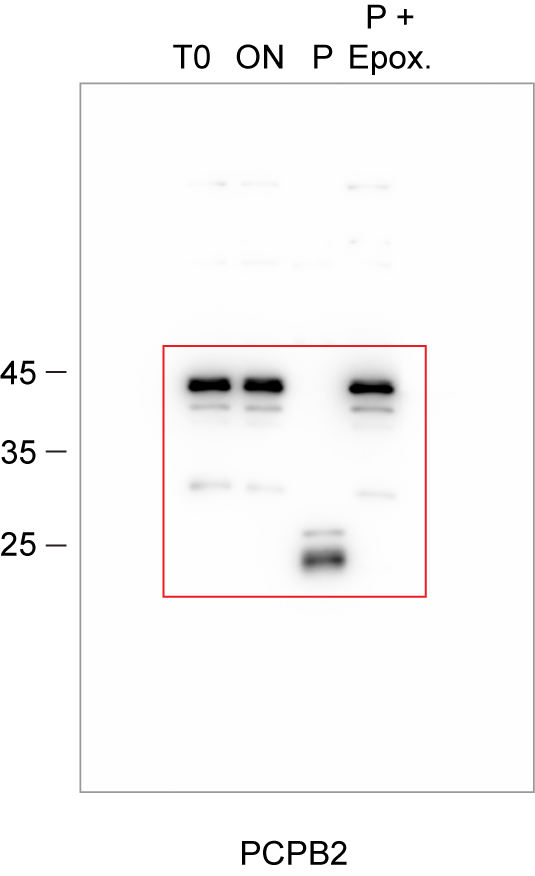

Supplement: Supplementary file 7 — Source Data Fig. 7 [file 44320_2024_15_MOESM7_ESM.zip › SD_Figure_7/7C Western blots/Fig 7C Western PCPB2.tif]

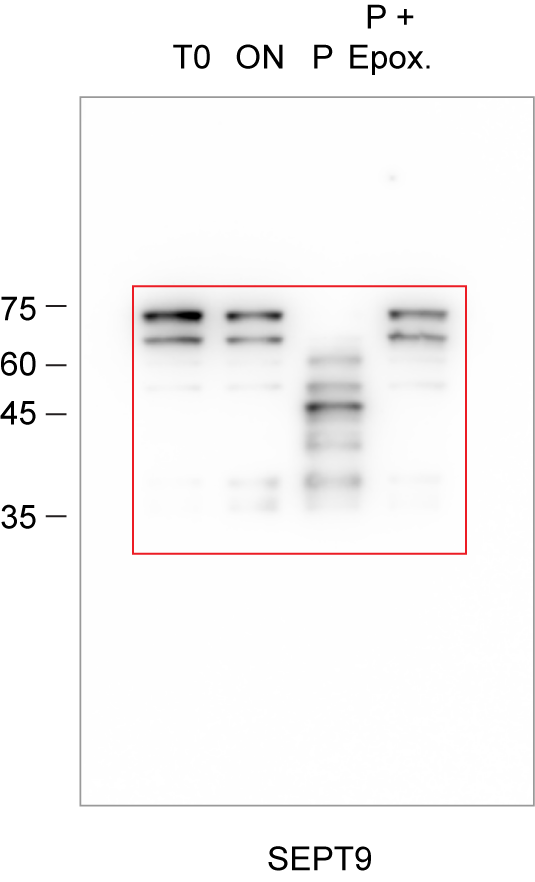

Supplement: Supplementary file 7 — Source Data Fig. 7 [file 44320_2024_15_MOESM7_ESM.zip › SD_Figure_7/7C Western blots/Fig 7C Western SEPT9.tif]

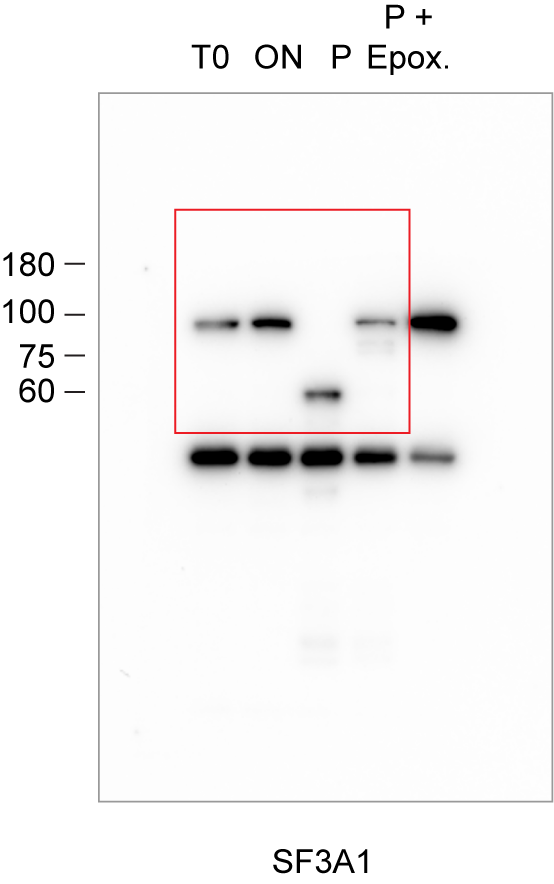

Supplement: Supplementary file 7 — Source Data Fig. 7 [file 44320_2024_15_MOESM7_ESM.zip › SD_Figure_7/7C Western blots/Fig 7C Western SF3A1.tif]

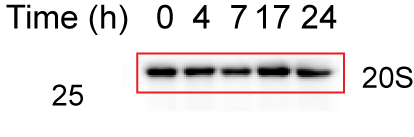

Supplement: Supplementary file 8 — Source Data for EV Figures [file 44320_2024_15_MOESM8_ESM.zip › SD_for_EV_FIgures/SD for Figure EV2/EV2B/EV2B Western 20S.tif]

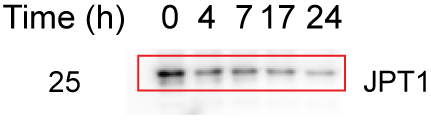

Supplement: Supplementary file 8 — Source Data for EV Figures [file 44320_2024_15_MOESM8_ESM.zip › SD_for_EV_FIgures/SD for Figure EV2/EV2B/EV2B Western JPT1.tif]

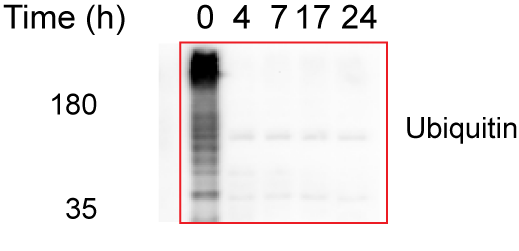

Supplement: Supplementary file 8 — Source Data for EV Figures [file 44320_2024_15_MOESM8_ESM.zip › SD_for_EV_FIgures/SD for Figure EV2/EV2B/EV2B Western Ubiquitin.tif]

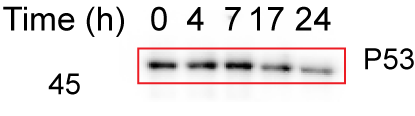

Supplement: Supplementary file 8 — Source Data for EV Figures [file 44320_2024_15_MOESM8_ESM.zip › SD_for_EV_FIgures/SD for Figure EV2/EV2B/EV2B Western P53.tif]

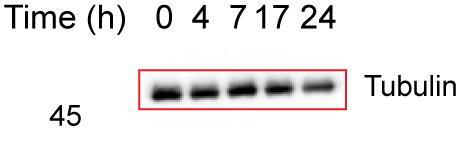

Supplement: Supplementary file 8 — Source Data for EV Figures [file 44320_2024_15_MOESM8_ESM.zip › SD_for_EV_FIgures/SD for Figure EV2/EV2B/EV2B Western Tubulin.tif]

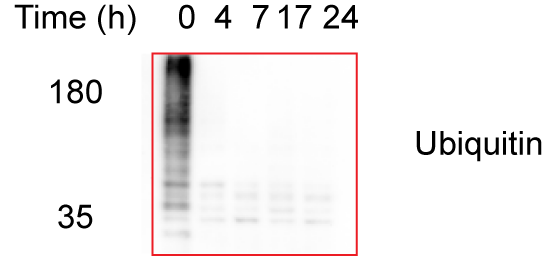

Supplement: Supplementary file 8 — Source Data for EV Figures [file 44320_2024_15_MOESM8_ESM.zip › SD_for_EV_FIgures/SD for Figure EV2/EV2C/EV2C Western Ubiquitin.tif]

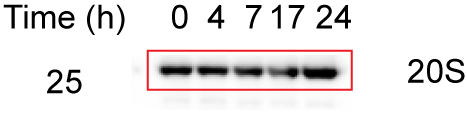

Supplement: Supplementary file 8 — Source Data for EV Figures [file 44320_2024_15_MOESM8_ESM.zip › SD_for_EV_FIgures/SD for Figure EV2/EV2C/EV2C Western 20S.tif]

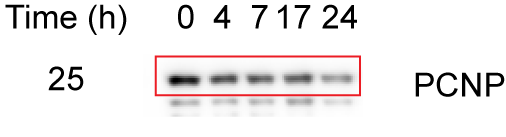

Supplement: Supplementary file 8 — Source Data for EV Figures [file 44320_2024_15_MOESM8_ESM.zip › SD_for_EV_FIgures/SD for Figure EV2/EV2C/EV2C Western PCNP.tif]

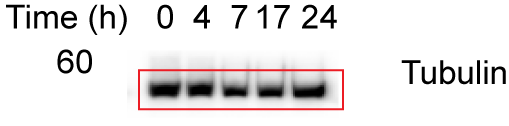

Supplement: Supplementary file 8 — Source Data for EV Figures [file 44320_2024_15_MOESM8_ESM.zip › SD_for_EV_FIgures/SD for Figure EV2/EV2C/EV2C Western Tubulin.tif]

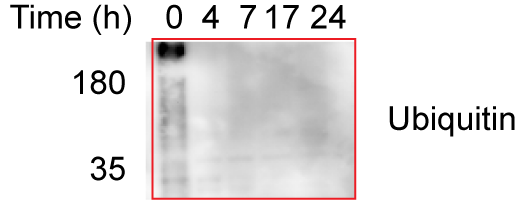

Supplement: Supplementary file 8 — Source Data for EV Figures [file 44320_2024_15_MOESM8_ESM.zip › SD_for_EV_FIgures/SD for Figure EV2/EV2A/EV2A Western Ubiquitin.tif]

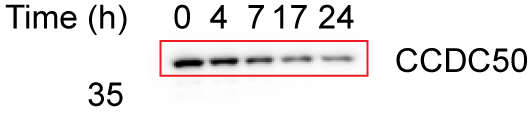

Supplement: Supplementary file 8 — Source Data for EV Figures [file 44320_2024_15_MOESM8_ESM.zip › SD_for_EV_FIgures/SD for Figure EV2/EV2A/EV2A Western CCDC50.tif]

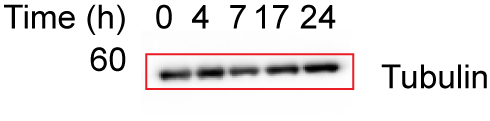

Supplement: Supplementary file 8 — Source Data for EV Figures [file 44320_2024_15_MOESM8_ESM.zip › SD_for_EV_FIgures/SD for Figure EV2/EV2A/EV2A Western Tubulin.tif]

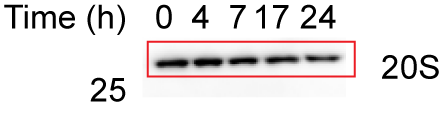

Supplement: Supplementary file 8 — Source Data for EV Figures [file 44320_2024_15_MOESM8_ESM.zip › SD_for_EV_FIgures/SD for Figure EV2/EV2A/EV2A Western 20S.tif]

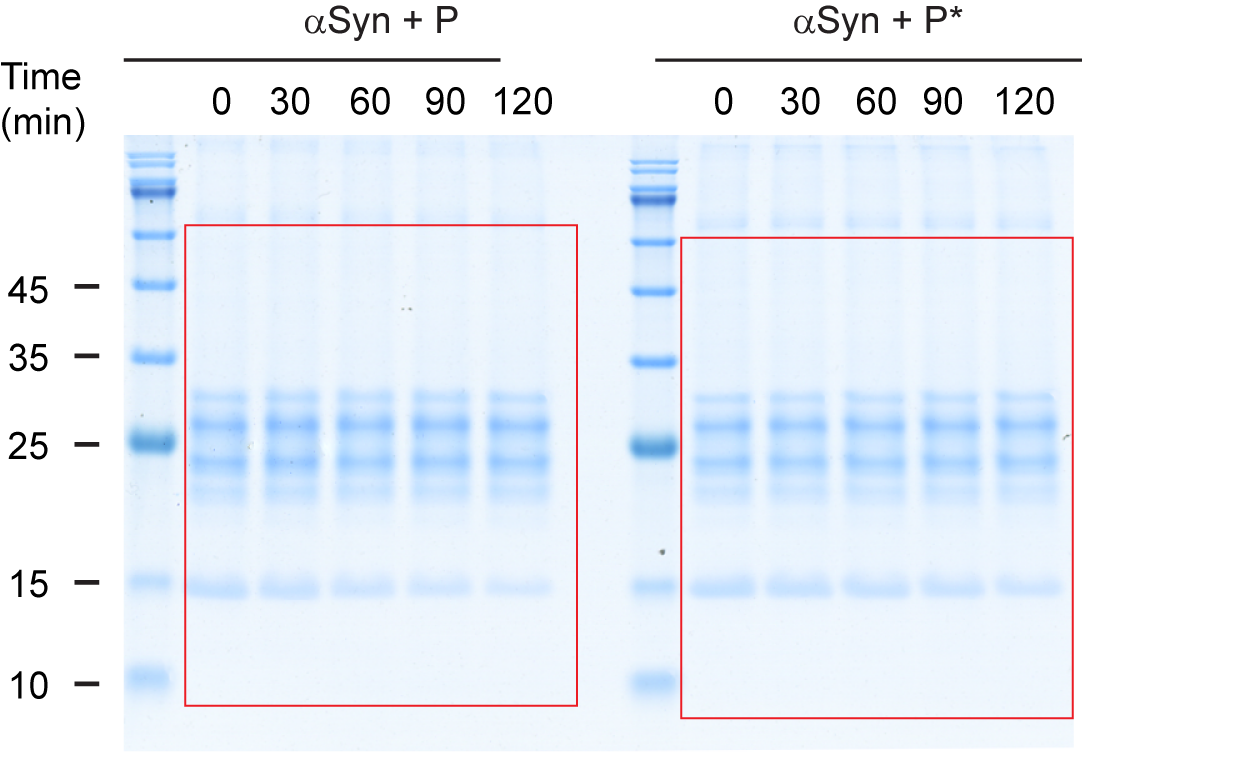

Supplement: Supplementary file 8 — Source Data for EV Figures [file 44320_2024_15_MOESM8_ESM.zip › SD_for_EV_FIgures/SD for Figure EV5/EV5A/EV5A Coomassie aSyn + P and Pstar.tif]

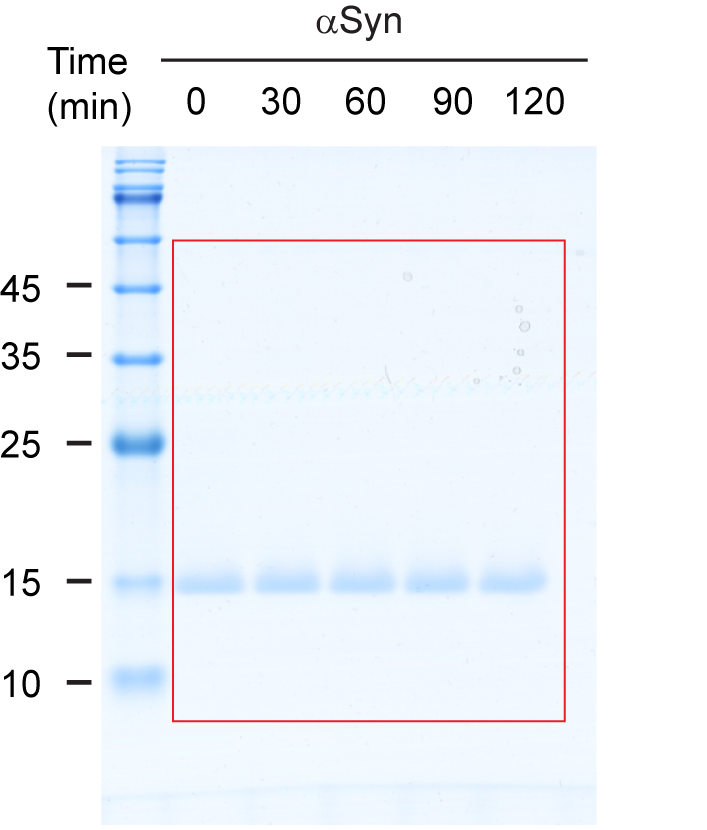

Supplement: Supplementary file 8 — Source Data for EV Figures [file 44320_2024_15_MOESM8_ESM.zip › SD_for_EV_FIgures/SD for Figure EV5/EV5A/EV5A Coomassie aSyn.tif]

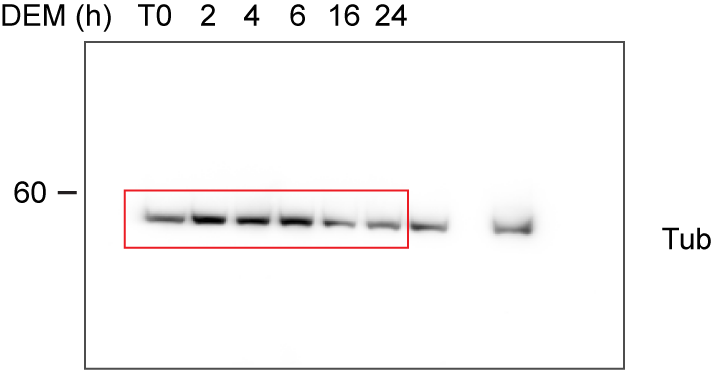

Supplement: Supplementary file 8 — Source Data for EV Figures [file 44320_2024_15_MOESM8_ESM.zip › SD_for_EV_FIgures/SD for Figure EV4/EV4A/EV4A Western Tubulin.tif]

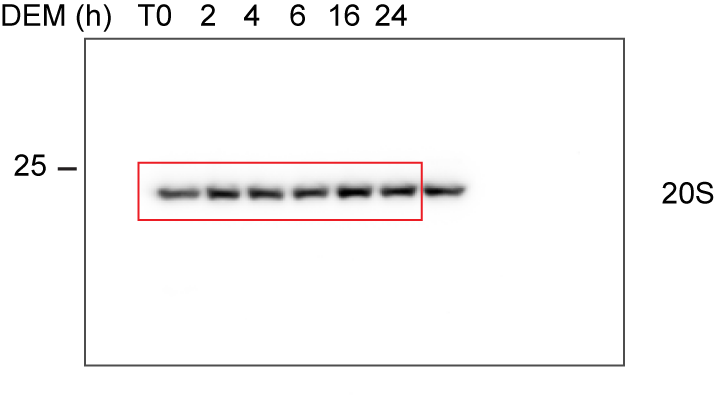

Supplement: Supplementary file 8 — Source Data for EV Figures [file 44320_2024_15_MOESM8_ESM.zip › SD_for_EV_FIgures/SD for Figure EV4/EV4A/EV4A Western 20S.tif]

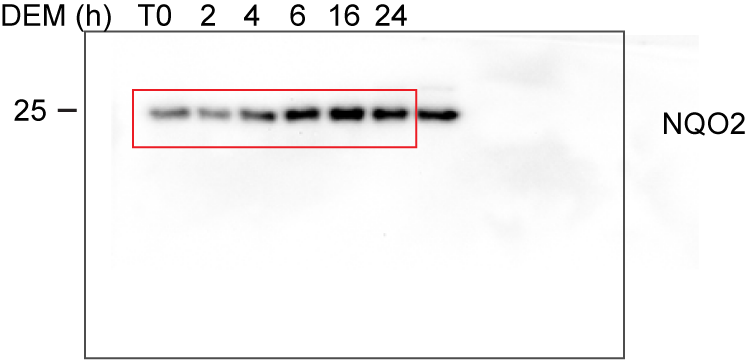

Supplement: Supplementary file 8 — Source Data for EV Figures [file 44320_2024_15_MOESM8_ESM.zip › SD_for_EV_FIgures/SD for Figure EV4/EV4A/EV4A Western NQO2.tif]

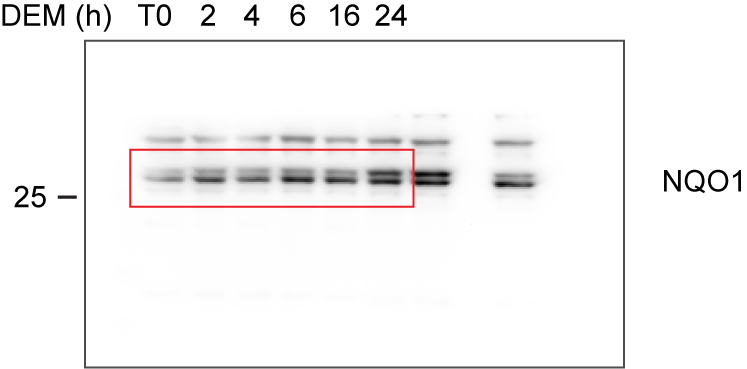

Supplement: Supplementary file 8 — Source Data for EV Figures [file 44320_2024_15_MOESM8_ESM.zip › SD_for_EV_FIgures/SD for Figure EV4/EV4A/EV4A Western NQO1.tif]
