# Supplementary material for: Systematic identification of 20S proteasome substrates
Source: Mol Syst Biol. 2024 Jan 29;20(4):403–27. doi: 10.1038/s44320-024-00015-y (PMC10987551; doi:10.1038/s44320-024-00015-y)
Supplement: Supplementary file 10 — Expanded View Figures [file 44320_2024_15_MOESM10_ESM.pdf]

Expanded View Figures

Figure EV1. Analysis of significantly changing peptides in PiP-MS.

(**A**) Volcano plot shows significantly changing peptides of proteasomal cleavage, followed by GluC degradation. Each point represents a peptide measured. The color indicates whether the change is indicative of reduction in abundance due to degradation (red), increase in abundance (dark gray) or no significant change (light gray). (**B**) String network (Szklarczyk et al, 2021) of proteins that showed increased abundance upon addition of proteasome (three or more significantly changing peptides). Most peptides originate from the proteasome complex. The lines show confident physical interactions (confidence cutoff >0.7). (**C**, **D**) The plot shows degradation of specific proteins selected for validation. Each line represents a peptide at a specific amino acid position. The color indicates whether the peptide showed significant signs of degradation (red and blue) or not (gray). Namely, peptides decreasing in abundance (dark red), specific peptides completely disappearing upon addition of proteasome (blue) or new semi-specific (proteasome-specific) peptides appearing (light red).

 © The Author(s)

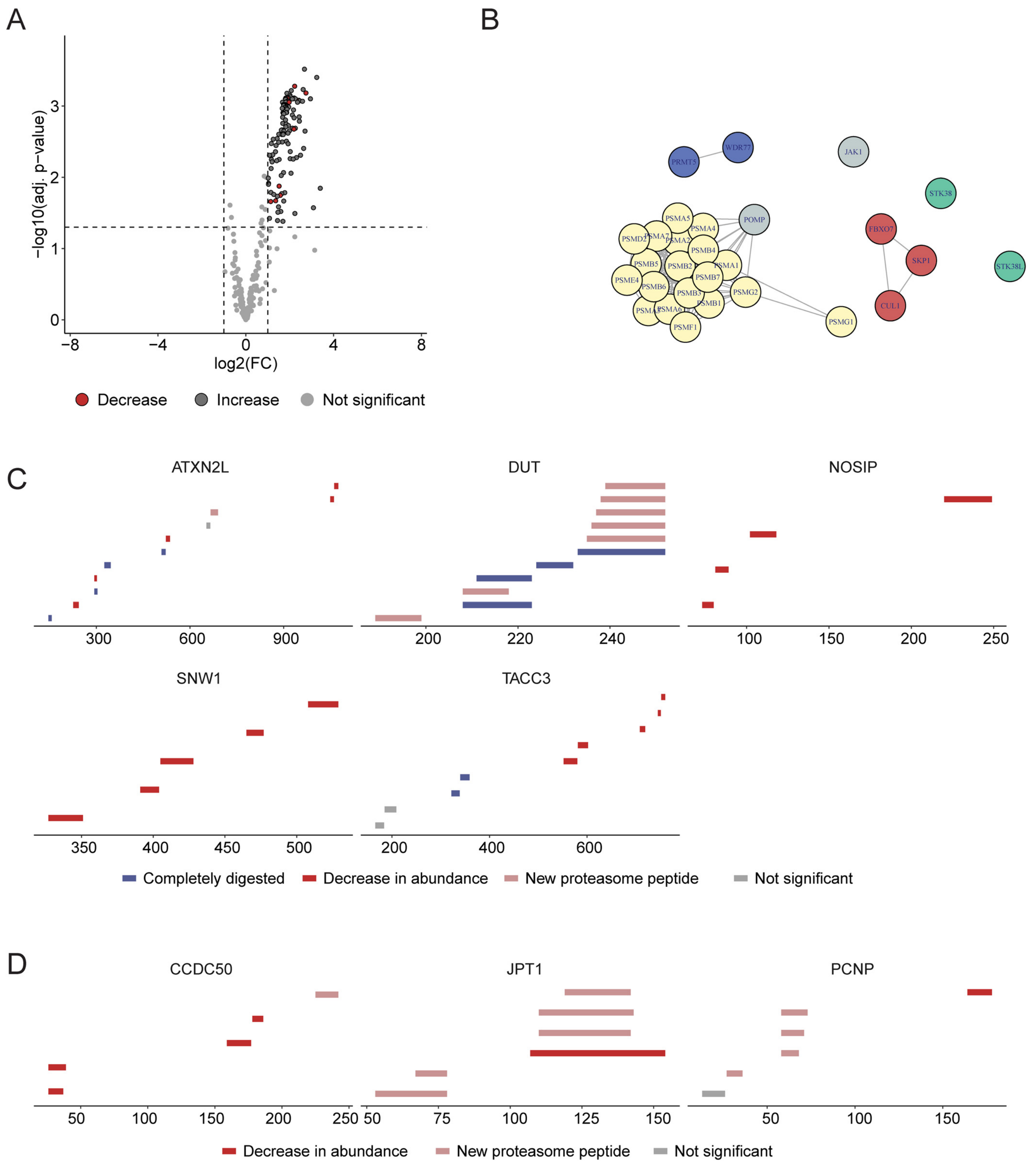
A
B
C
D
−log10(adj. p−value)
log2(FC)
Decrease
Increase
Not significant
PRMT5
WDR77
JAK1
STK38
POMP
FBXO7
SKP1
CUL1
STK38L
PSMG1
PSMG2
PSMF1
ATXN2L
DUT
NOSIP
SNW1
TACC3
Completely digested
Decrease in abundance
New proteasome peptide
Not significant
CCDC50
JPT1
PCNP
Decrease in abundance
New proteasome peptide
Not significant

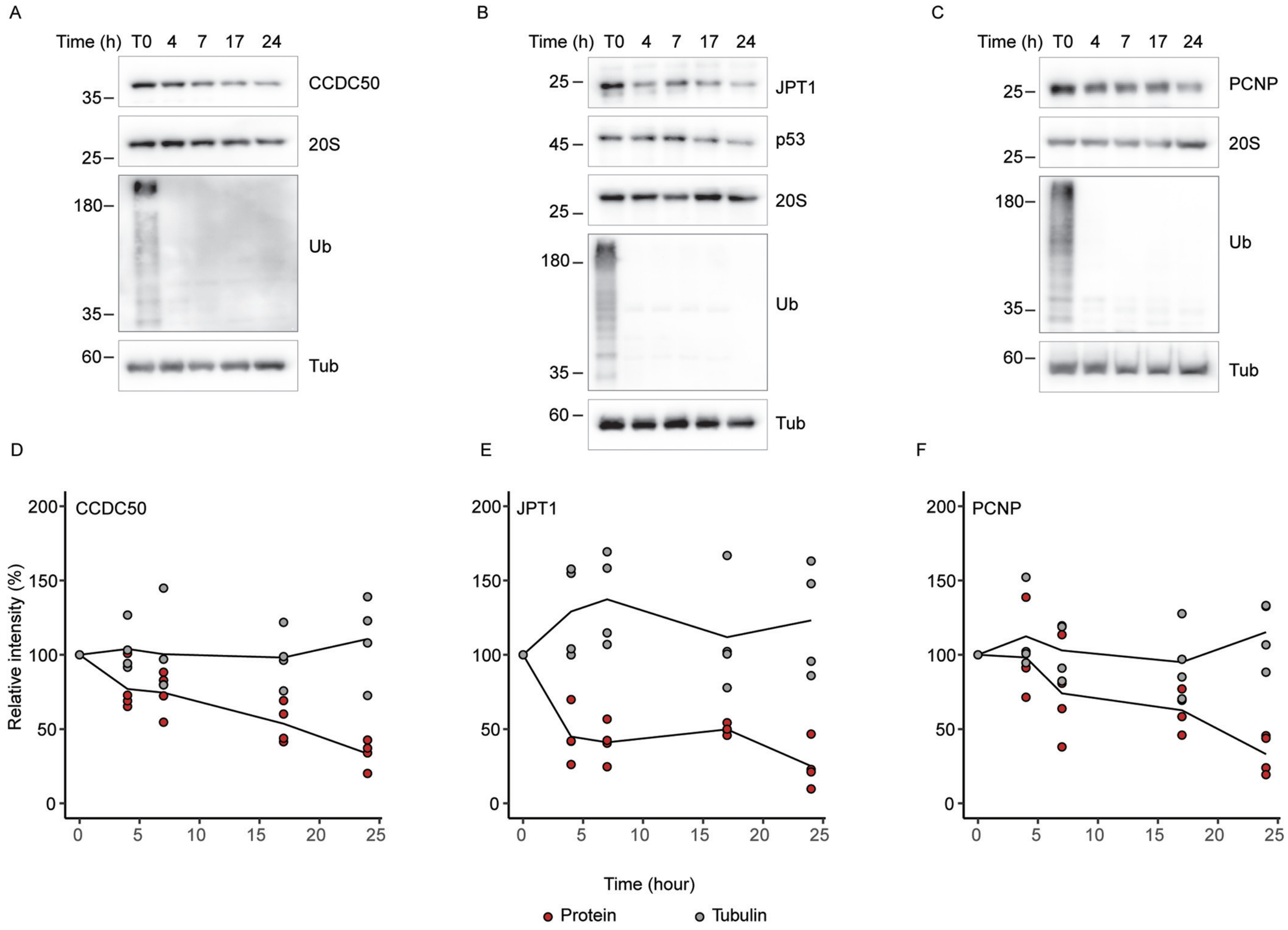

Figure EV2. Cycloheximide chase assay for monitoring the cellular stability of the PiP-MS hits.

Representative western blots showing the 20S proteasome-dependent degradation of (**A**) CCDC50, (**B**) JPT1 and (**C**) PCNP. Cells were treated with the ubiquitination inhibitor, TAK-243 together with cycloheximide, to inhibit protein synthesis. Cells were harvested after 4, 7, 17 and 24 h. Stabilities of the target proteins (FLAG-tagged) and the 20S proteasome were analyzed by western blots using antibodies against FLAG, p53 and PSMA1 (a 20S proteasome subunit). To monitor the inactivation of the ubiquitination cascade an anti-ubiquitin antibody was used. p53 and Tubulin (Tub) were used a positive and negative controls, respectively. Changes in the levels of the (**D**) CCDC50, (**E**) JPT1 and (**F**) PCNP relative to the initial time point were quantified from four independent experiments. Each point corresponds to an individual experiment, with red dots indicating the analyzed protein and gray dots representing tubulin, the control. Source data are available online for this figure.

© The Author(s)

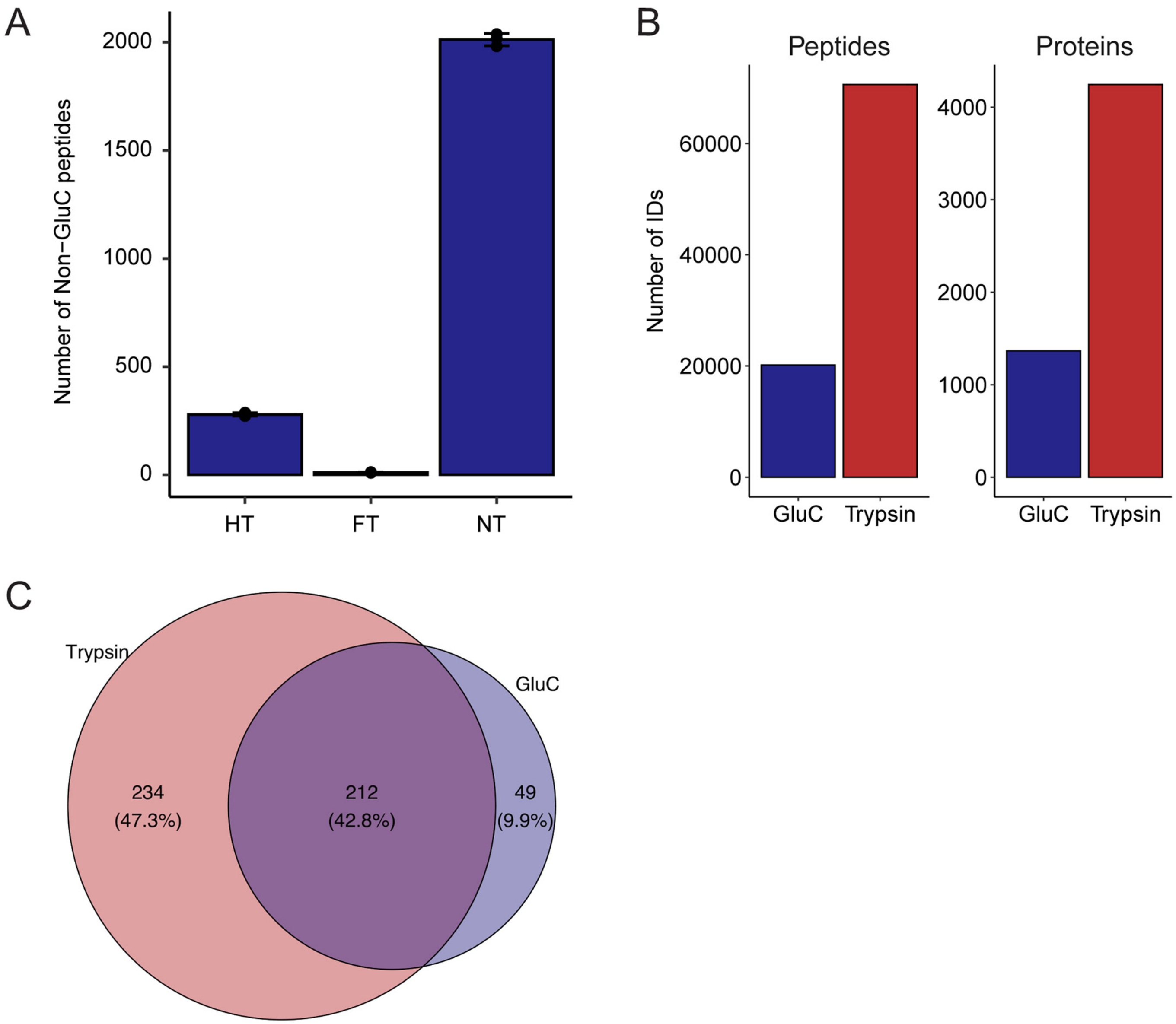

Figure EV3. Assessing the efficiency of the second digestion step using GluC or trypsin.

(**A**) Analysis of proteasome unique peptides in the GluC experiment reveal that most peptides have no tryptic ends (NT). Fully-tryptic (FT) peptides have two tryptic ends, and half-tryptic (HT) peptides have one tryptic end. Error bars represent the mean $+/-$ SD of $n = 3$ replicates. (**B**) Number of detected peptides and proteins when Trypsin or GluC was used in the second processing step of PiP-MS experiment. (**C**) Number of degraded proteins identified with two different enzymes. Only proteins detected in both datasets were considered in this analysis.

© The Author(s)

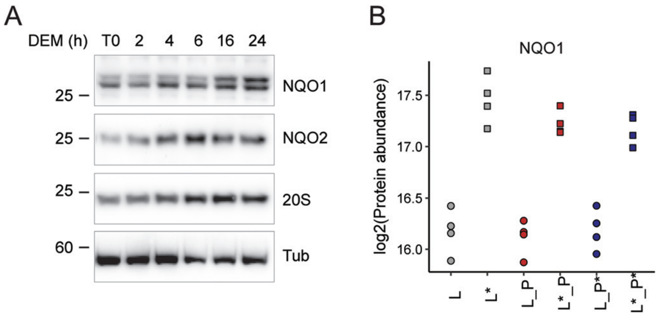

Figure EV4. Induction of oxidative stress by treating cells with DEM.

(**A**) Following the exposure of cells to DEM, western blot analysis was used for monitoring the levels of NQO1, NQO2 and an 20S proteasome subunit (20S, PSMA1). Tubulin (Tub) served as a control. As expected, induction of oxidative stress led to an increase in NQO1, NQO2 and 20S proteasome levels. The experiment was repeated three times. (**B**) LC/MS proteomic analysis similarly indicates an upregulation of NQO1 under conditions of oxidative stress (labeled with an asterisk for Lysate (L) and Proteasome (P)) relative to naïve conditions. Each point corresponds to an individual experiment. Source data are available online for this figure.

© The Author(s)

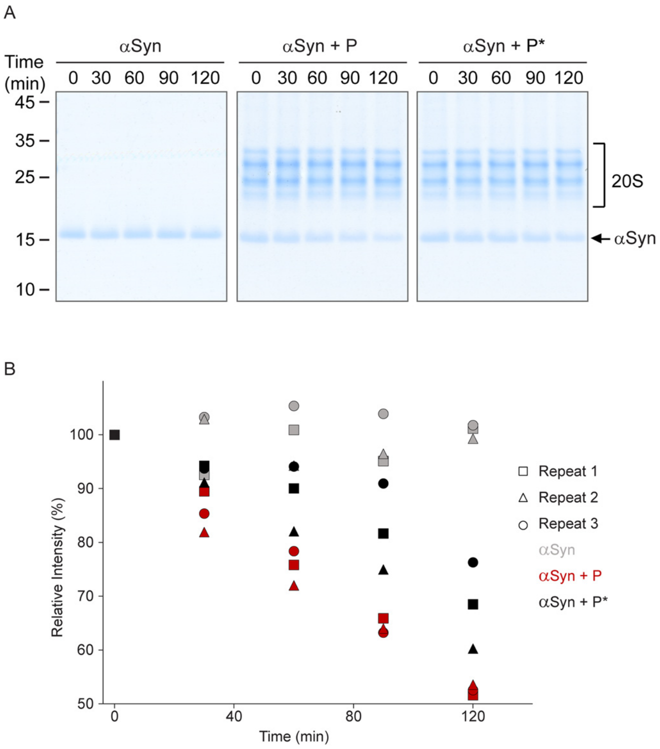

Figure EV5. The naïve proteasome more efficiently degrades α-synuclein compared to its oxidized counterpart.

(**A**) Representative time-dependent degradation assays of α-synuclein (αSyn) in the presence of naïve (P) and oxidized (P*) 20S proteasomes. As a control, the stability of α-synuclein was measured in the absence of the proteasome. (**B**) Raw data for Fig. 4C. Squares, triangles and circles represent biological repeats. Gray, red and black data points denote the levels of α-synuclein (αSyn) over time in the absence or presence of naïve (P) and oxidized (P*) 20S proteasomes. Source data are available online for this figure.

© The Author(s)
